# Application of virtual reality haptic simulators in periodontics: insights from dental professionals

**DOI:** 10.3389/froh.2025.1719790

**Published:** 2025-12-15

**Authors:** Santiago Arias-Herrera, Zaraida Catala-Oriola, Joao Firmino-Canhoto, Anabel Gramatges-Rojas, María Florencia Sittoni-Pino, Nicla Flacco

**Affiliations:** Faculty of Health Sciences, Department of Preclinical Dentistry, Universidad Europea de Valencia, Valencia, Spain

**Keywords:** haptic virtual reality, dental education, prophylaxis, manual dexterity, preclinicaltraining, periodontics

## Abstract

**Introduction:**

Virtual reality haptic simulator (VRHS)-training devices that integrate visual immersion with tactile feedback- are increasingly used in dental education, yet their application in periodontics remains limited. Sonic scaling requires high tactile precision and control, making it a suitable domain for evaluating VRHS efficacy. This study assessed faculty perceptions and performance using VRHS for preclinical periodontal training compared with conventional simulation.

**Methods:**

A quasi-experimental, repeated-measures study was conducted with 30 calibrated faculty members (15 periodontists; 15 general dentists) from the European University of Valencia. Each participant performed standardized calculus removal tasks using both the Simodont® dental trainer and conventional mannequin-based models. Objective outcomes—treatment time, residual calculus, and tip angulation—were recorded, and perceptions were collected through a 14-item Likert questionnaire and open-ended questions adapted from Philip et al. (2023) and Bakr et al. (2016).

**Results:**

Treatment time was significantly shorter in conventional training (26.7% completed <2 min) compared with VRHS (13.3%; *p* = 0.033). VRHS achieved higher residual calculus scores (3.00 ± 0.00 vs. 2.75 ± 0.21; *p* < 0.001) and improved tip angulation (2.68 ± 0.36 vs. 2.51 ± 0.44; *p* = 0.015). Within VRHS, periodontists outperformed general dentists in angulation (2.85 ± 0.18 vs. 2.52 ± 0.42; *p* = 0.010), though no other group differences were significant. Satisfaction scores were high (mean = 4.16 ± 0.45), and internal consistency was acceptable (Cronbach's *α* = 0.73). Faculty valued repetition, feedback, and confidence building but noted limited realism—particularly the absence of water, soft tissues, and subgingival calculus.

**Conclusion:**

VRHS improved precision and consistency in preclinical scaling while requiring longer completion times. Faculty endorsed its educational value as a complementary tool to conventional training. Continued refinement in tactile realism and broader multicenter validation are needed to consolidate its integration into periodontal education.

## 1 Introduction

Correct hand-feel during sonic instrumentation is fundamental for achieving safe and effective periodontal debridement. Sonic devices require precise control of angulation, lateral pressure, and movement to generate the desired cavitation and shockwave effects. However, the high-frequency vibration of these instruments makes it difficult for novice learners to perceive subtle tactile cues, which complicates the acquisition of proper psychomotor skills (1). To overcome these challenges, intensive faculty supervision and repeated live-action training are essential during preclinical practice. Yet, many dental schools lack structured curricula or specialized training protocols dedicated to sonic instrumentation (2).

In response to these deficiencies, a variety of surrogate training models—such as quail eggs, pop-top cans, and bionic replicas of extracted teeth—have been incorporated into preclinical periodontal education (3, 4). While these traditional models offer some didactic value, they do not accurately replicate the tactile sensations or complex feedback required for mastering sonic scaling. Virtual simulation (VS) technologies, by contrast, have the potential to provide realistic, repeatable, and standardized training opportunities with real-time feedback, thereby supporting self-directed learning and sustainable skill development (5–9).

Recent advancements in robotics, haptics, and virtual reality (VR) have further expanded the potential of simulation-based dental education. Modern VR haptic simulators (VRHS) allow students to practice critical psychomotor skills—such as hand-eye coordination, fine motor control, and mirror-assisted instrumentation—within controlled and resource-efficient environments. Haptic feedback, in particular, enables the reproduction of tactile sensations that approximate real clinical procedures, enhancing the authenticity of simulation-based learning. Consequently, dental schools worldwide are increasingly integrating VRHS into their preclinical curricula to facilitate the transition from simulation laboratories to patient care settings (10).

Haptic integration has been successfully applied across multiple medical and dental disciplines, improving training, diagnostic accuracy, and procedural performance. For instance, in surgery, haptics-enhanced simulators allow trainees to rehearse minimally invasive procedures with realistic tactile fidelity. In dentistry, the use of VRHS in restorative training has been widely reported, with students frequently valuing its ability to provide immediate feedback and a safe environment for early skills acquisition (11). At the same time, limitations are also acknowledged: students often report reduced realism in patient interaction and tactile sensation compared to training on typodonts or real patients (12, 13). These findings suggest that VRHS should be considered as a complementary tool rather than a full substitute for traditional training modalities, underscoring the need for hybrid approaches in comprehensive dental education.

Despite the growing body of evidence in restorative dentistry and endodontics, limited research has investigated the role of VRHS in other key fields such as periodontics, anaesthesia, and implantology (7, 14–17). Moreover, the literature to date has largely focused on the perceptions of preclinical students, while the perspectives of dental faculty—who play a critical role in curriculum design, supervision, and skill assessment—remain underexplored (10, 18, 19). Addressing this gap is particularly important in periodontics, where mastering the subtle psychomotor skills of sonic scaling is both technically demanding and pedagogically challenging (4, 20). Therefore, the objective of this study was to evaluate the potential impact of VRHS on the teaching of sonic scaling techniques in preclinical periodontics training, specifically from the perspective of dental faculty. Furthermore, the study aimed to identify perceived strengths and weaknesses of VRHS in this context. We hypothesized that the integration of VRHS into preclinical periodontics training for sonic scaling procedures would be perceived by faculty as an effective adjunct to conventional methods, with repeated exposure reinforcing its pedagogical value while also revealing specific limitations.

## 2 Materials and methods

### 2.1 Study design

This study is classified as a quasi-experimental prospective repeated-measures design in simulation, reported according to STROBE-SBR recommendations for simulation-based research (21), and complemented by CROSS/CHERRIES for the survey component (22, 23). The procedural protocols applied across the different experimental conditions, together with the anonymized rubric scores and questionnaire dataset, are provided as Supplementary Materials accompanying this article.

### 2.2 Participants

The study population included 184 faculty members from the Department of Dentistry at European University of Valencia (Spain). The eligible population comprised those faculty members who were general dentists routinely performing periodontal treatments, as well as specialists in periodontics whose clinical practice was dedicated primarily or exclusively to periodontal care. Additional inclusion criteria required participants to have at least a basic level of competence in the use of haptic simulators for training purposes and a minimum of three years of experience in preclinical teaching within the Dentistry degree program. Exclusion criteria comprised faculty members who were not licensed dentists, dentists whose primary clinical practice was devoted to other dental specialties, and faculty members not actively engaged in teaching duties at the time of the study. Faculty participants were specifically selected because their teaching expertise and curricular responsibilities allowed for a critical evaluation of the VR-Haptic simulator's pedagogical potential, ensuring that their insights could inform curriculum design, teaching methodology, and student assessment approaches. These criteria were applied to ensure a homogeneous sample of participants with comparable baseline expertise in both conventional and VRHS-based preclinical training modalities. Once all eligible faculty members were identified, they were provided with comprehensive information about the study, including its objectives, scope, potential benefits and risks, and the voluntary nature of participation. Confidentiality of responses and data anonymization procedures were explicitly guaranteed. Faculty members who agreed to participate and signed the informed consent form constituted the final study population.

**Figure 1:**
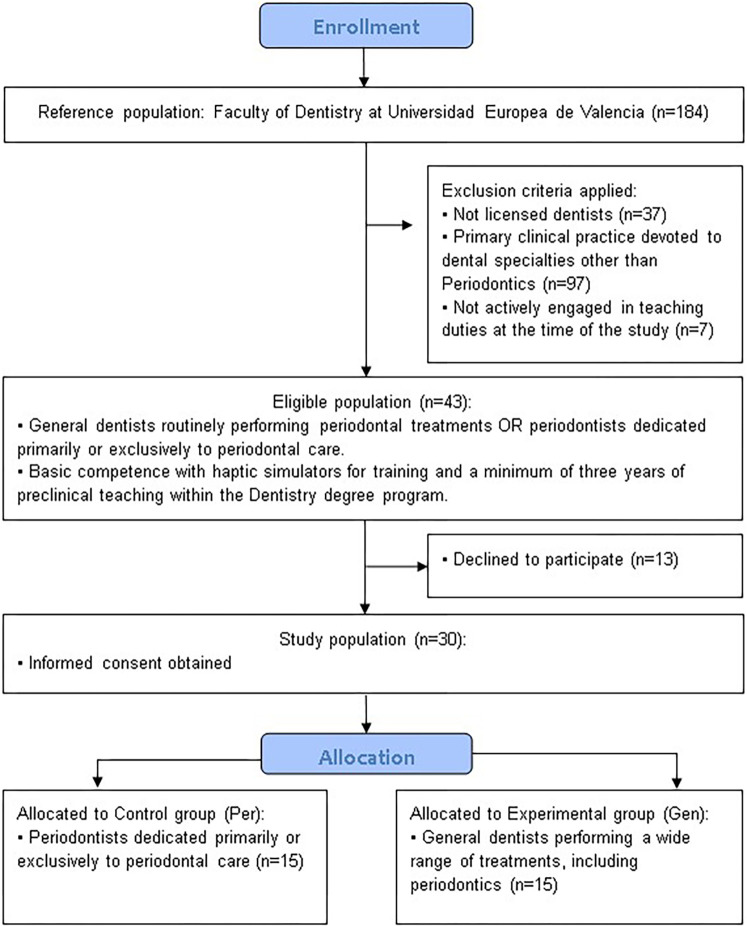
Experimental flow diagram summarizing the study design.

The faculty members who consented to participate and constituted the study population were categorized into two groups according to their professional profile. The control group (Per) consisted of periodontists whose clinical practice was dedicated primarily or exclusively to periodontal care, while the experimental group (Gen) included general dentists routinely performing a wide range of dental treatments, including but not limited to periodontics. This stratification allowed the comparison of perceptions between specialists and non-specialists regarding the integration of VRHS in preclinical periodontal training (Figure 1).

### 2.3 Intervention

One week before the intervention, the entire study population underwent a calibration session using the Simodont® dental trainer (SDT) (Nissin Dental Products Europe B.V., Nieuw-Vennep, Netherlands.) to ensure a minimum skill baseline of 75% in periodontal instrumentation tasks. This session lasted approximately 15 min and was conducted at the SmartLab of Universidad Europea de Valencia under the supervision of two experts in VRHS training (S.A. and F.S.). The purpose of this step was to standardize participants' familiarity with the simulator and establish comparable baseline competence prior to data collection.

The calibration began with the *Manual Dexterity Training module*, in which participants practiced fundamental psychomotor skills such as pressure control, fine motor coordination, and tactile sensitivity. In these standardized exercises, the objective was to remove a predefined target volume from virtual objects while minimizing contact with the surrounding safety margin. The simulator provided immediate visual and haptic feedback, and work assessment was generated automatically. To qualify for the study, participants were required to achieve a minimum score of 75%, reflecting accurate target removal with limited errors in adjacent areas.

After completing the manual dexterity tasks, participants proceeded to the *Scaling Calibration Module*, performing standardized calculus removal exercises on the anterior mandibular region (teeth 31, 41). The tasks required correct adaptation and angulation of the sonic scaler tip, execution of short overlapping strokes, and consistent application of light lateral pressure to detach calculus deposits without damaging the root surface. The simulator automatically generated performance scores based on the percentage of calculus removed. Participants were required to achieve at least 75% successful calculus removal with less than 5% root surface loss in order to be considered calibrated.

This structured calibration ensured that all faculty members were equally familiar with the Simodont® environment and had attained a comparable baseline level of manual skill and haptic sensitivity before progressing to the experimental intervention.

Calibration procedures were only required for the VRHS environment, as participants were already proficient in conventional preclinical training methods. All faculty members had extensive prior experience with sonic scaling on mannequin-based simulators, which forms part of the standard curriculum in preclinical dentistry. Given this background and the straightforward nature of the procedure, additional calibration on the conventional simulators was not deemed necessary (Figure 1).

#### 2.3.1 Haptic virtual reality simulation training method

**Figure 2:**
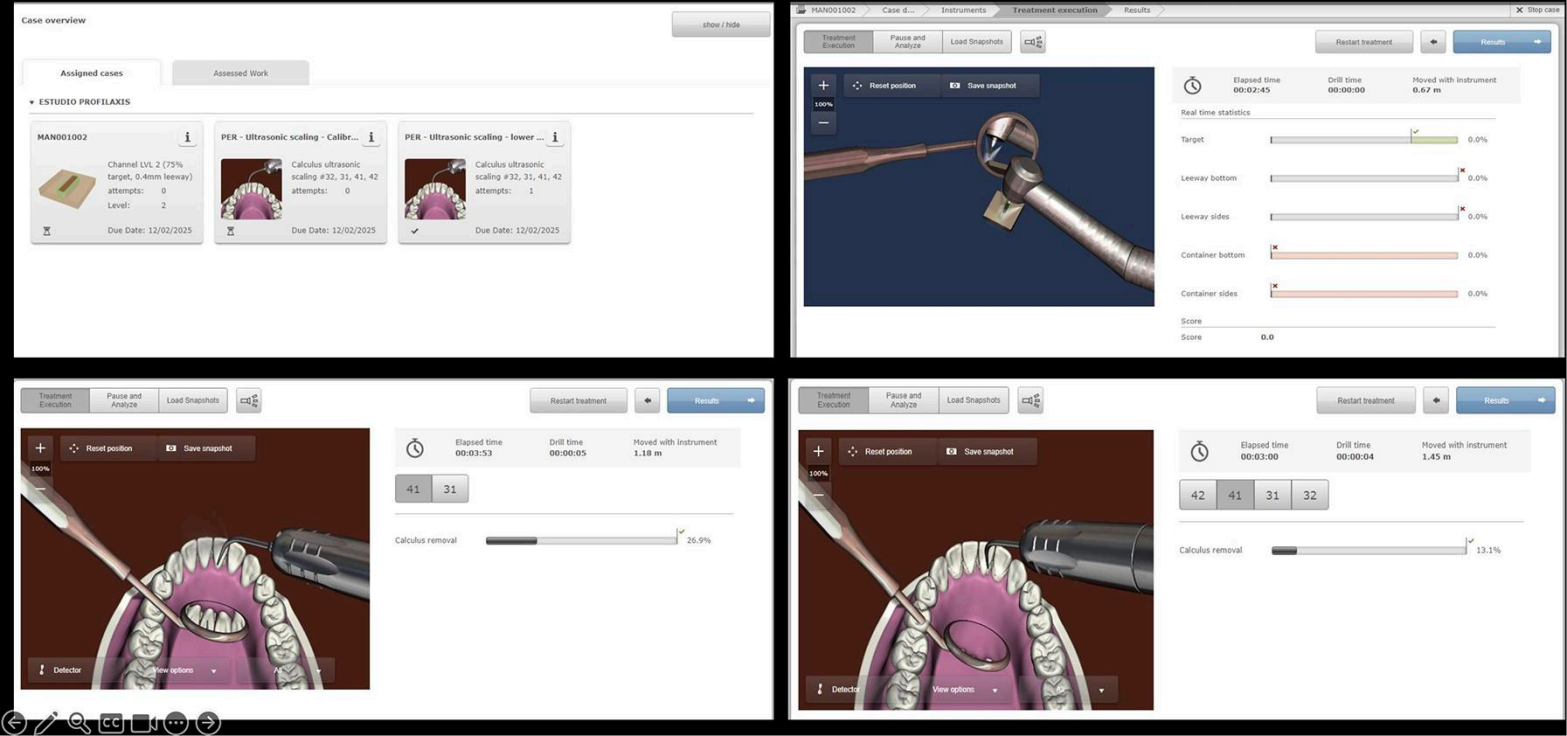
Schematic representation of the virtual reality haptic simulator (VRHS) interface and training modules used for sonic scaling practice.

During the first experimental session, all participants performed calculus removal procedures on the lower anterior teeth using the SDT. The virtual model employed was the Nissin-700HPRO_proto_wcal v18 in the mandibular anterior region (teeth 31, 32, 41, 42), the most up-to-date periodontology model available from Nissin®. The simulator generated an objective calculus removal score per tooth, reflecting the percentage of deposits successfully eliminated (Figure 2). Participants were instructed to achieve the highest possible score within the allotted time frame, focusing on correct tip adaptation, angulation, and stroke control.

#### 2.3.2 Conventional preclinical training method

In the second session, the same participants performed the equivalent procedure on conventional simulators. These consisted of mannequin heads with rubber cheeks fitted with periodontology-specific maxillary and mandibular models (Frasaco AP-Z, Tettnang, Germany) with elastic, exchangeable gingival layers (A-PZ WOK and A-PZ WUK) and screw-retained single-rooted teeth with artificial calculus positioned at teeth 31, 32, 41, and 42 (A-PZ ZEC 1). All procedures were conducted using standard preclinical equipment, including suction devices and the NSK AS2000 air scaler (NSK, Kanuma, Tochigi Prefecture, Japan) fitted with the S3 Z252412 tip scaler (NSK, Kanuma, Tochigi Prefecture, Japan). No additional calibration was performed on conventional simulators, as all participants were experienced in preclinical mannequin-based training and routinely engaged in sonic scaling instruction, ensuring homogeneity of baseline competence for this modality.

#### 2.3.3 Survey component

Following completion of both training sessions, participants completed a structured questionnaire designed to capture sociodemographic data and perceptions of VRHS use in preclinical periodontal education. The instrument was primarily adapted from the validated survey by Philip et al. (19), with selected items incorporated from Bakr et al. (24) to reinforce comparative dimensions (e.g., realism, motivation, and VRHS vs. conventional training).

The final questionnaire comprised 14 items organized into four thematic blocks: satisfaction and confidence, perceived educational value, comparative perceptions, and qualitative insights. Content validity was established through expert panel review and pilot testing with a subset of faculty not included in the main study. Details of the adaptation process, validation procedure, and complete questionnaire are provided in Supplementary Table S1.

### 2.4 Variables

The primary exposure in this study was participation in two simulation-based training conditions: (i) virtual reality haptic simulation (VRHS) using the Simodont® dental trainer, and (ii) conventional mannequin-based simulation using Frasaco periodontal models with sonic devices. The rationale for this design was to evaluate the potential of VRHS as a complementary educational tool, given its capacity to provide automated and objective scoring, in contrast to conventional methods where evaluation relies on examiner judgment.

**Primary outcomes**: Periodontal treatment quality was assessed through three predefined technical criteria recorded in both simulation modalities:

• Time: total time required to complete calculus removal, recorded automatically in the VRHS and with a stopwatch in the conventional simulator. Time was categorized as fast (≤2 min), moderate (2–5 min), or slow (≥5 min).
• sonic tip positioning: tip angulation and parallelism to the root surface were assessed for all tooth surfaces (M, D, B, L) by two calibrated investigators, applying the same 3-point rubric in both simulation modalities: 1 **=** perpendicular to the root surface, 2 = oblique, 3 = parallel. Mean values across the four surfaces were used for analysis. Amount of calculus removed: in the VRHS, the system automatically provided the percentage of deposit elimination per surface. For comparability with the conventional simulator, these percentages were converted into an equivalent 3-point ordinal scale: 1 = <50% calculus removed, 2 = 50%–95% calculus removed, 3 = >95% calculus removed. In the conventional simulator, examiners applied the same categorical rubric based on visual inspection of calculus removal: 1 = entire surface covered with calculus, 2 = some residual calculus, 3 = no calculus.

**Secondary outcomes**: Following both sessions, participants completed a structured questionnaire capturing sociodemographic data and perceptions of VRHS. The final instrument included 14 items organized into four thematic blocks (satisfaction and confidence; perceived educational value; comparative perceptions; qualitative insights), adapted from Philip et al. (19) and Bakr et al. (24) (see Supplementary Table S1). Of these, 10 items were closed-ended statements rated on a 5-point Likert scale (*1* *=* *Strongly Disagree*, *2* *=* *Disagree*, *3* *=* *Neutral*, *4* *=* *Agree*, *5* *=* *Strongly Agree*), and 4 items were open-ended questions designed to elicit qualitative reflections on the perceived strengths, limitations, and pedagogical value of the VRHS experience.

The first three thematic domains (*satisfaction and confidence; perceived educational value; comparative perceptions*) corresponded to the Likert-scale items, while the fourth domain (*qualitative insights*) included the open-ended questions. The instrument was reviewed for clarity and content validity by three dental education experts and pilot-tested before administration to ensure comprehension and internal consistency.

**Calibration of examiners**: For the conventional simulator assessments, two examiners (J.F. and Z.C.) underwent a calibration process prior to data collection. Ten standardized scaling procedures were independently scored at two time points after a two-week interval, and intra- and inter-examiner agreement was calculated using Cohen's Kappa to ensure reliability of the rubric (25–27).

**Potential confounders and effect modifiers** considered in the study design included participants' clinical background (periodontist vs. general dentist), prior experience with preclinical teaching, and the fixed order of exposure (VRHS followed by conventional simulation), which could introduce learning or fatigue effects. To minimize confounding, all participants underwent calibration on the VRHS system prior to the intervention to establish a standardized minimum competence level, and conventional training was not calibrated further given the participants' extensive prior experience in preclinical mannequin-based teaching. Examiner variability was addressed through a calibration process and reliability testing using Cohen's Kappa coefficient.

### 2.5 Data sources and measurement

All assessments were conducted in the SmartLab of the European University of Valencia. Performance in the VRHS condition was automatically recorded by the Simodont® dental trainer (time, and calculus removal), while tip position was recorded manually by two calibrated examiners. Likewise, the same examiners manually performed the measurements in the conventional condition (time, tip position and calculus removal) using a structured rubric. Examiner reliability was verified through Cohen's Kappa. The perception questionnaire was adapted from previously validated instruments (19, 24), and underwent expert panel review and pilot testing to ensure content validity. To minimize potential sources of bias, all participants received standardized calibration in VRHS prior to the study, conventional training was not recalibrated due to participants' extensive prior experience, and examiner assessments were blinded to participants' specialty profile.

### 2.6 Sample size

The sample size was estimated *a priori* using G*Power version 3.1 (Heinrich Heine University, Düsseldorf, Germany) based on a paired-samples design for repeated measures. In the absence of previous studies with similar outcomes, a large effect size (Cohen's *d* = 0.8), a significance level of 0.05 (two-tailed), and a statistical power of 80% were assumed. Under these parameters, a minimum total of 30 participants was required. Given that 184 faculty members constituted the eligible population, all were invited to participate in order to maximize representativeness and account for potential non-response.

### 2.7 Statistical methods

Residual calculus and tip angulation were originally rated on 3-point ordinal scales by two independent evaluators for each tooth. Evaluator ratings were averaged to obtain a single mean score per tooth and participant; subsequently, for each participant, scores were averaged across teeth 42, 41, 31, and 32 to derive composite measures for each training modality (VRHS and conventional). Although the underlying scales were ordinal, the repeated measurements and aggregation justified treating the composite outcomes as approximately continuous for inferential analyses.

Descriptive statistics were computed for all variables and are reported as mean ± standard deviation (SD) for continuous data and as frequencies and percentages for categorical data. Distributional assumptions were examined with the Shapiro–Wilk test; homogeneity of variances was checked with Levene's test where applicable.

Group comparisons in Table 1 (demographics) used Student's *t*-test for continuous variables and the chi-square test (or Fisher's exact test when expected counts were <5) for categorical variables. For treatment time (three ordered categories), within-subject comparisons between VRHS and conventional modalities used the Wilcoxon signed-rank test, and between-group comparisons within each modality (periodontists vs. general dentists) used chi-square or Fisher's exact tests.

**Table 1:** Demographic and professional characteristics of the participants.

Variable	Per (*n* = 15)	Gen (*n* = 15)	*p*-value	Effect size (Cohen’s *d* [95% CI])
Age (years)	41.6 ± 7.7	38.2 ± 6.7	0.208	0.47 [–0.26, 1.19]
Sex, *n* (%)				
Male	7 (46.7%)	3 (20.0%)	0.121	—
Female	8 (53.3%)	12 (80.0%)		
Educational level, *n* (%)				
Bachelor’s degree	1 (6.7%)	3 (20.0%)	0.270	—
Master’s degree	6 (40.0%)	8 (53.3%)		
Doctorate	8 (53.3%)	4 (26.7%)		
Clinical experience (years)	18.8 ± 8.7	15.3 ± 8.0	0.256	0.42 [–0.33 to 1.14]
Teaching experience (years)	11.5 ± 7.8	10.3 ± 7.2	0.648	0.17 [–0.57 to 0.90]
Teaching experience in periodontology (years)	4.3 ± 3.2	0.3 ± 0.5	<0.001**	1.73 [0.82 to 2.60]

Values are presented as mean ± standard deviation or *n* (%). Between-group comparisons were performed using independent-samples *t*-tests (Levene's test for equality of variances) or *χ*^2^ tests as appropriate. Effect sizes (Cohen’s *d* [95% Confidence Interval]) are reported for continuous variables to indicate the magnitude of differences between groups*. p* < 0.05 was considered statistically significant.

Per, periodontists; Gen, general dentists.

**Indicates that the result is highly significant.

For the composite performance outcomes (residual calculus and tip angulation), within-subject comparisons between modalities used the Wilcoxon signed-rank test. Between-group comparisons within each modality used Student's *t*-test for independent samples or the Mann–Whitney *U* test when parametric assumptions were not satisfied. To assess the combined effect of professional group and training modality, two-way mixed ANOVA models were fitted with Group (periodontist vs. general dentist) as the between-subjects factor and Modality (VRHS vs. conventional) as the within-subjects factor (sphericity is not applicable with two levels). Effect sizes were reported as partial eta-squared $\left(\eta_{p}^{2}\right)$ for ANOVA and Cohen's d for pairwise comparisons.

Responses to the 10-item satisfaction questionnaire (5-point Likert scale) were analysed descriptively, and internal consistency was evaluated using Cronbach's *α*. A global satisfaction score was calculated for each participant as the mean of all items; between-group comparisons for the global score (and, exploratorily, for individual items) were performed using the Mann–Whitney *U* test.

The responses to the open-ended questions were analysed manually, in order to identify key concepts common to each question. This approach allows the quantification of common perceptions, even if described by the faculty with different words.

All analyses were conducted in IBM SPSS Statistics, version 29 (IBM Corp., Armonk, NY, USA). Two-tailed *p*-values <0.05 were considered statistically significant.

## 3 Results

### 3.1 Participant flow

A total of 184 faculty members from the Faculty of Dentistry at Universidad Europea de Valencia were initially considered (reference population). Of these, 141 were excluded for not meeting the inclusion criteria (37 were not licensed dentists, 97 had their primary clinical practice devoted to specialties other than Periodontics, and 7 were not actively engaged in teaching duties at the time of the study). The eligible population therefore comprised 43 dentists.

**Figure 3:**
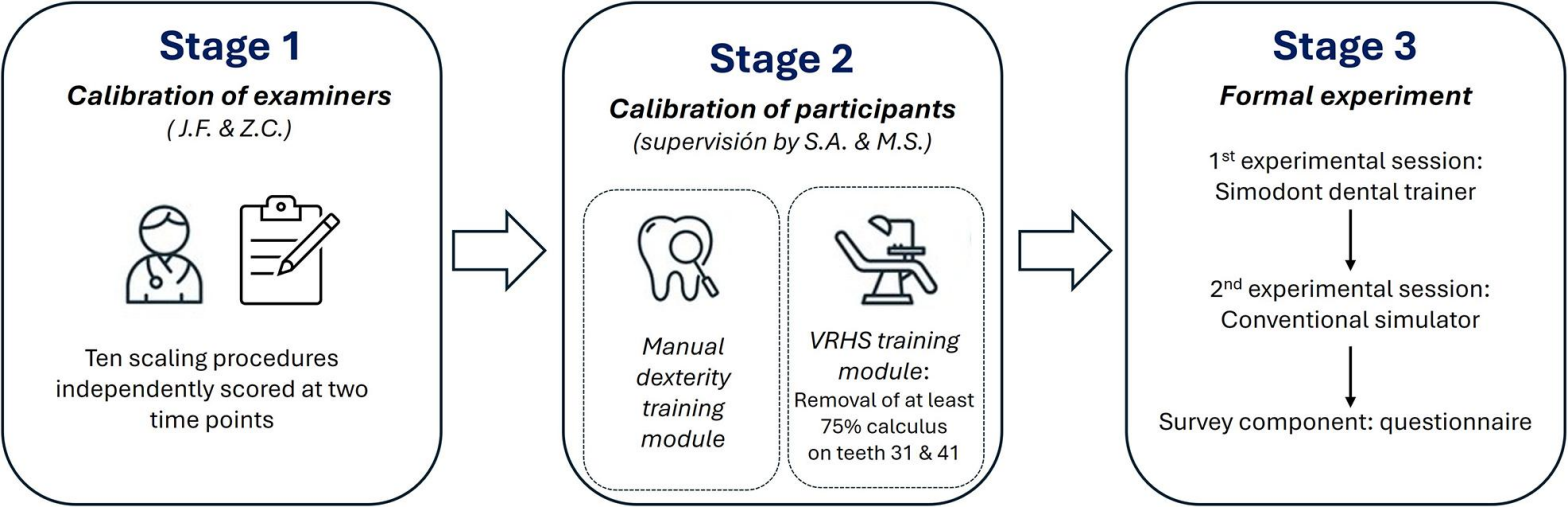
Flow chart of participant selection and allocation throughout the study.

Among them, 13 declined to participate, resulting in a study population of 30 dentists who provided informed consent. Participants were allocated to the control or experimental group according to their clinical experience. The control group (*n* = 15) was composed of periodontists dedicated primarily or exclusively to periodontal care, whereas the experimental group (*n* = 15), consisted of general dentists routinely performing a wide range of treatments, including periodontics. All participants received the assigned intervention and were included in the final analysis (Figure 3).

### 3.2 Baseline characteristics

The demographic and professional characteristics of the participants are summarized in Table 1. The sample included 30 educators (10 men, 20 women; mean age 39.9 ± 7.3 years, range 28–56). Periodontists and general dentists did not differ significantly in age, overall clinical experience, or general teaching experience. The distribution of sex and educational level was also comparable between groups. As expected, periodontists reported significantly greater teaching experience specifically in periodontology (*p* < 0.001).

### 3.3 Primary outcomes

#### 3.3.1 Treatment time

The distribution of treatment times is shown in Table 2. Overall, treatment times differed significantly between VRHS and conventional training (Wilcoxon signed-rank test, *Z* = 2.14, *p* = 0.033, *r* = 0.39). Conventional training was associated with a higher proportion of procedures completed in less than 2 min (26.7% vs. 13.3%), whereas longer durations (>5 min) occurred only in the VRHS modality.

**Table 2:** Distribution of treatment time categories by training modality and professional groups.

Treatment time	VRHS (*n* = 30)	CONV (*n* = 30)	VRHS—Per (*n* = 15)	VRHS—Gen (*n* = 15)	CONV—Per (*n* = 15)	CONV—Gen (*n* = 15)
>5 min	4 (13.3%)	0 (0.0%)	1 (6.7%)	3 (20.0%)	0 (0.0%)	0 (0.0%)
2–5 min	22 (73.3%)	22 (73.3%)	12 (80.0%)	10 (66.7%)	12 (80.0%)	10 (66.7%)
<2 min	4 (13.3%)	8 (26.7%)	2 (13.3%)	2 (13.3%)	3 (20.0%)	5 (33.3%)
Statistic	*Z* *=* *2.14, p* *=* *0.033*		*χ*^2^(2) = 1.18, *p* = 0.55		Fisher, *p* = 0.68	
	*Effect size (r* *=* *0.39)*					

Treatment time was recorded in three ordinal categories: *>* 5 min, 2–5 min, and <2 min. Values are expressed as frequency and percentage of cases in each category. Comparisons between training modalities (VRHS = Virtual Reality Haptic Simulation, CONV = conventional training) were performed using the Wilcoxon signed-rank test and the effect size was calculated as *r* = *Z*/√*N*. Comparisons between professional groups (Per vs. Gen s)were performed by chi-square test or Fisher’s exact test.

VRHS, Virtual Reality Haptic Simulation; CONV, conventional training; Per, periodontists; Gen, general dentists.

When stratified by professional group, no significant differences were observed between periodontists and general dentists. In the VRHS modality, the distribution across the threetime categories (>5 min, 2–5 min, <2 min) did not differ significantly between groups [*χ*^2^(2) = 1.18, *p* = 0.55]. Similarly, in the conventional modality, treatment times were comparable between groups (Fisher's exact test, *p* = 0.68).

#### 3.3.2 Residual calculus and tip angulation by tooth

Table 3 presents the comparison of residual calculus and sonic tip angulation between modalities across the four evaluated teeth. Residual calculus scores were consistently higher in VRHS compared with conventional instrumentation. In VRHS, evaluators uniformly assigned the maximum score (3 = no residual calculus), while in the conventional modality some variability was observed, with partial calculus persistence (score 2) in several cases. The Wilcoxon signed-rank test confirmed statistically significant differences favouring VRHS for each tooth (all *p* < 0.001), with large effect size (*r* = 0.65–0.71).

**Table 3:** Comparison of residual calculus and sonic tip angulation between VRHS and CONV instrumentation across teeth.

Tooth	Residual calculus (Mean ± SD)			Tip angulation (Mean ± SD)		
	VRHS	CONV	*p-value/r*	VRHS	CONV	*p-value*
*42*	3.00 ± 0.00	2.73 ± 0.28	<0.001**/0.71	2.66 ± 0.37	2.46 ± 0.48	0.041*/0.37
*41*	3.00 ± 0.00	2.79 ± 0.23	<0.001**/0.68	2.68 ± 0.36	2.52 ± 0.48	*0.052/0.35*
*31*	3.00 ± 0.00	2.73 ± 0.30	<0.001**/0.65	2.71 ± 0.39	2.56 ± 0.46	0.046*/0.36
*32*	3.00 ± 0.00	2.75 ± 0.26	<0.001**/0.70	2.67 ± 0.41	2.50 ± 0.47	*0.053/0.35*

Residual calculus was rated on a 3-point ordinal scale (1 = entire surface covered with calculus; 2 = some residual calculus; 3 = no calculus). Each value represents the mean of three independent evaluator ratings per tooth. Tip angulation was assessed on a 3-point scale (1 = perpendicular; 2 = oblique; 3 = parallel). Each value represents the mean of two independent evaluator ratings per tooth. Data are presented as mean ± standard deviation. Paired comparisons between VRHS (virtual reality haptic simulator) and CONV (conventional)instrumentation were performed using the Wilcoxon signed-rank test (*n* = 30). The effect size (r) was calculated as *r* *=* *Z/√N.*

VRHS, Virtual Reality Haptic Simulation; CONV, conventional training.

*Indicates that the result is statistically significant.

**Indicates that the result is highly significant.

Tip angulation scores showed a different pattern. For teeth 42 and 31, VRHS training resulted in significantly more parallel positioning of the sonic tip compared with conventional training (*p* = 0.041 and *p* = 0.046, respectively), corresponding to medium effect sizes (*r* = 0.35–0.37). Differences for teeth 41 (*p* = 0.052, *r* = 0.35) and 32 (*p* = 0.053, *r* = 0.34) did not reach statistical significance.

#### 3.3.3 Composite scores of residual calculus and tip angulation

Table 4 summarizes the composite measures across teeth 42, 41, 31, and 32. For residual calculus, scores were significantly higher in VRHS compared with conventional instrumentation (3.00 ± 0.00 vs. 2.75 ± 0.21; *p* < 0.001; *r* = 0.82). All participants consistently achieved the maximum score in the VRHS modality, indicating complete calculus removal without variability among evaluators. This ceiling effect precluded further between-group comparisons within VRHS. In the conventional modality, some residual deposits remained, but no significant differences were observed between periodontists and general dentists (2.76 ± 0.21 vs. 2.73 ± 0.21; *p* = 0.355). The Group × Modality interaction was not significant (*p* = 0.710, *η*^2^*_p_* = 0.005), indicating a negligible interaction effect.

**Table 4:** Composite scores of residual calculus and sonic tip angulation by professional group within VRHS and conventional training.

Variable	VRHS (*n* = 30)	CONV (*n* = 30)	Per—VRHS (*n* = 15)	Gen—VRHS (*n* = 15)	Per—CONV (*n* = 15)	Gen—CONV (*n* = 15)	*p (Group* *×* *Modality)*
Residual calculus	3.00 ± 0.00	2.75 ± 0.21	3.00 ± 0.00	3.00 ± 0.00	2.76 ± 0.21	2.73 ± 0.21	*p: 0.710*
	*p: <0.001*		—		*p: 0.355*		Partial *η*^2^: 0.005
	*r* *=* *0.82*						
Tip angulation	2.68 ± 0.36	2.51 ± 0.44	2.85 ± 0.18	2.52 ± 0.42	2.58 ± 0.33	2.43 ± 0.53	*p: 0.172*
	*p: 0.015*		p: 0.010*		*p: 0.193*		Partial *η*^2^: 0.066
	*r* *=* *0.42*						

Values represent the mean of evaluator ratings across teeth 42, 41, 31, and 32, averaged for each participant to obtain a composite score. Data are shown as mean ± standard deviation. Comparisons between training modalities (VRHS vs. CONV) were performed using the Wilcoxon signed-rank test, and the effect size (r) was calculated as *r* = *Z/√N.* Between-group comparisons (Per vs. Gen) within each modality were performed using Student’s *t*-test (or Mann–Whitney *U* test when non-normal distribution was observed). The Group × Modality interaction was tested with a mixed-effects ANOVA and the partial *η*^2^ was reported as a measure of effect size. A *p*-value < 0.05 was considered statistically significant.

VRHS, Virtual Reality Haptic Simulation; CONV, conventional training; Per, periodontists; Gen: general dentists.

*Indicates that the result is statistically significant.

**Figure 4:**
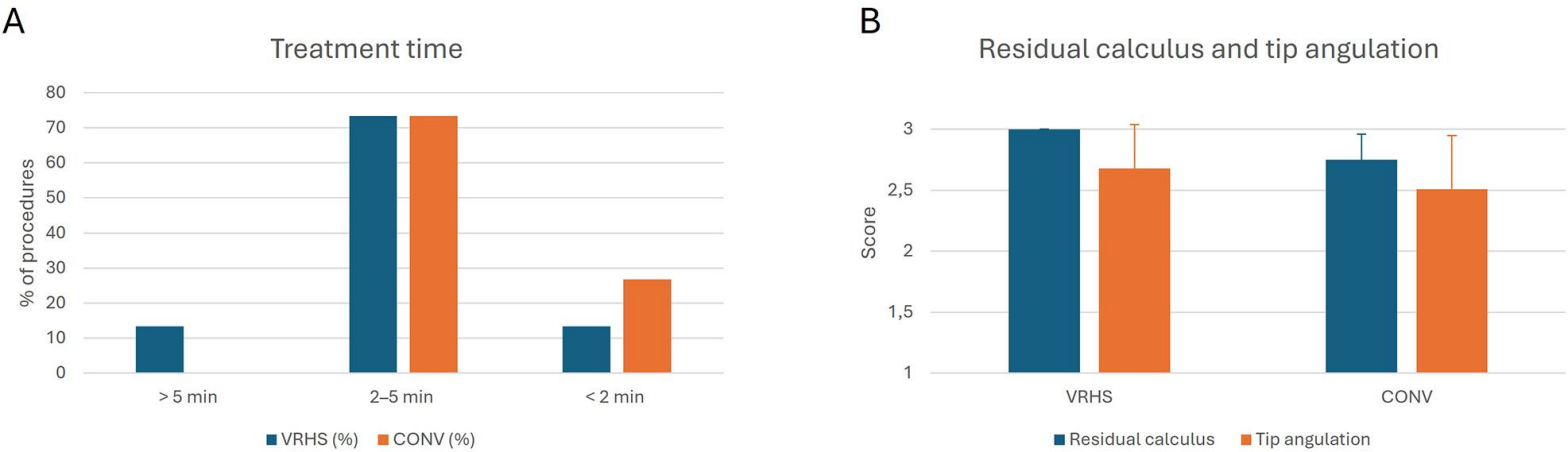
Objective performance outcomes comparing VRHS and CONV. **(A)** Distribution of treatment time categories across modalities. **(B)** Comparison of composite scores for residual calculus and sonic tip angulation (mean ± SD) in VRHS and CONV. *VRHS, Virtual Reality Haptic Simulation; CONV, conventional training.*

For tip angulation, VRHS was also associated with significantly higher scores compared with conventional training (2.68 ± 0.36 vs. 2.51 ± 0.44; *p* = 0.015, *r* = 0.42). Within the VRHS condition, periodontists achieved significantly higher scores than general dentists (2.85 ± 0.18 vs. 2.52 ± 0.42; *p* = 0.010), whereas no significant difference was detected in the conventional modality (2.58 ± 0.33 vs. 2.43 ± 0.53; *p* = 0.193). The Group × Modality interaction did not reach statistical significance (*p* = 0.172, *η*^2^*_p_* = 0.066), suggesting a moderate but nonsignificant interaction effect (Figure 4).

### 3.4 Secondary outcomes

#### Ten- item satisfaction questionnaire

**Figure 5:**
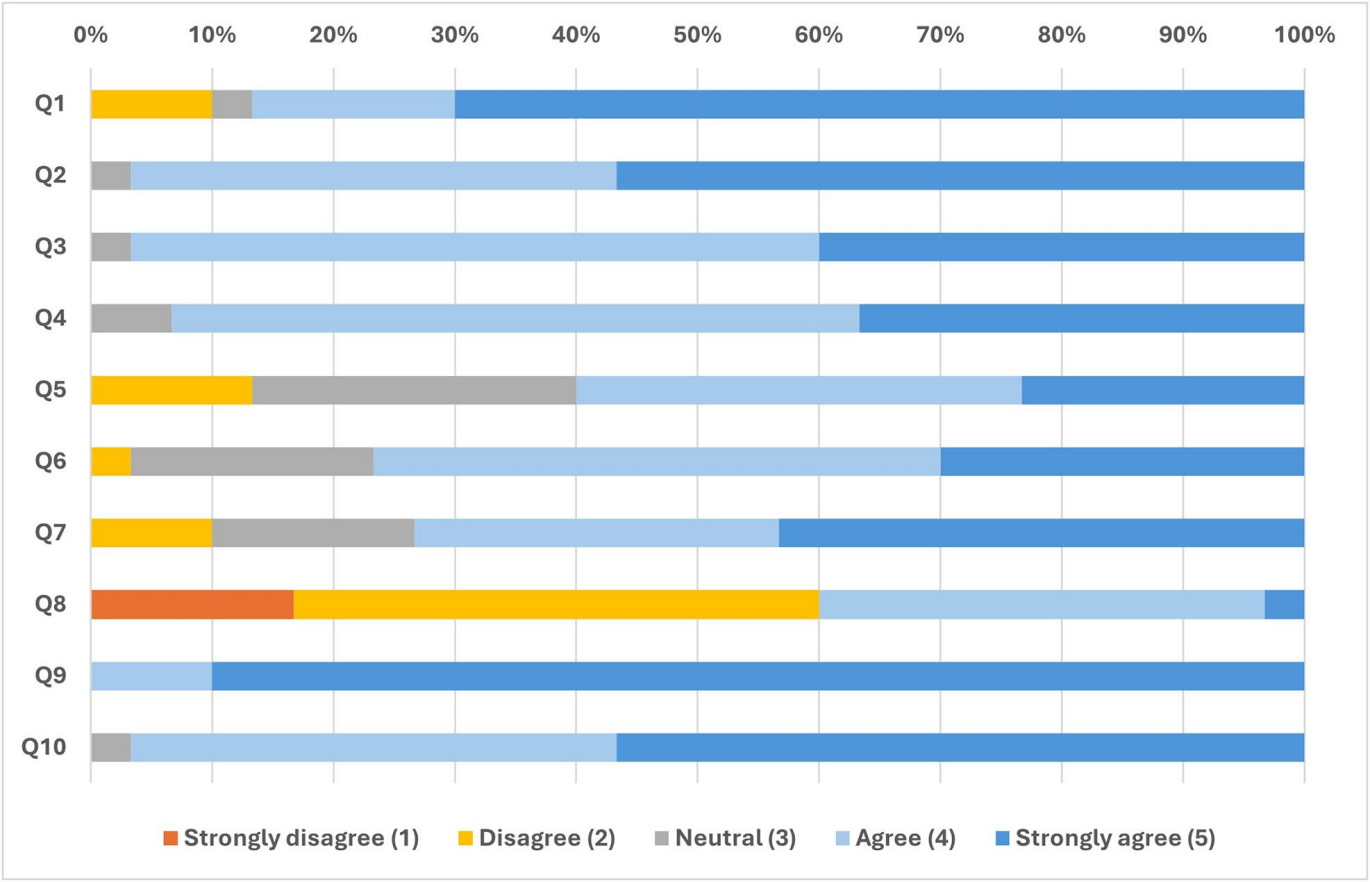
Distribution of responses to the 10-item satisfaction questionnaire**.** Stacked bars represent the percentage of participants selecting each response category on a 5-point Likert scale (1 = Strongly disagree; 5 = Strongly agree). Each bar corresponds to one questionnaire item (Q1–Q10, *n* = 30).

The distribution of responses to the 10-item satisfaction questionnaire is illustrated in Figure 5. Descriptive statistics for each item are provided in Supplementary Table S1. Overall, responses were skewed toward the higher end of the scale, with most items receiving median scores of 4 (“Agree”) or 5 (“Strongly agree”). The internal consistency of the questionnaire was acceptable (Cronbach's *α* = 0.727). Most items showed corrected item–total correlations above the recommended 0.30 threshold, except for Q2 (0.006) and Q8 (0.122), which were weakly associated with the overall scale. Deleting Q2 marginally improved internal consistency (*α* = 0.748), while deletion of Q8 produced only a minor increase (*α* = 0.718). Therefore, all items were retained for subsequent analyses.

A global satisfaction score was calculated for each participant as the mean of all 10 items. Periodontists reported a mean score of 4.11 ± 0.51, while general dentists scored 4.21 ± 0.38. The Mann–Whitney *U* test revealed no significant difference between groups (*U* = 125.0, *p* = 0.62). Full descriptive statistics are provided in Supplementary Table S2.

#### Open-ended questions

All but one faculty member answered the open-ended questions about the participants' experience of the VRHS learning environment. Due to the nature of the questions some answers were short and concise, while others pointed to more than one benefit or limitation.

Regarding the benefits associated with the utilization of the Simodont dental trainer, the predominant responses pertained to the capacity for repetition and the concomitant enhancement of trust and security inherent to that repetition. Repetition was cited on eight occasions, while trust and security were also referenced on eight separate instances. The enhancement in dexterity was noted by four respondents, while the tactile sensation (associated with pressure on the instrument) and the realism of the procedure received three mentions each. The concept of ergonomics was referenced on two occasions, and ultimately, the versatility of the Simodont dental trainer emerged as the most significant benefit for two faculty members, as it facilitates its application in diverse scenarios and enables the adaptation of the procedure to the individual student's proficiency level.

The most frequently cited limitation of the Simodont dental trainer was its lack of realism (mentioned 19 times), either by itself or in conjunction with the absence of water (four mentions), soft tissues (three mentions), blood, saliva, texture, aspiration, and subgingival calculus (one mention each). The lack of realism was also associated with discrepancies in tactile sensations and mobility of the virtual model (2 mentions), vibration, and limitations on the use of the intraoral mirror (1 mention). Other limitations of the Simodont included the restriction of access to the trainer itself (three mentions) and the absence of interaction with the patient (one mention). The absence of sensations (pain) was mentioned by two respondents, and the cost of the trainers, as well as the reduced number of exercises or model varieties, was mentioned three times.

The third question inquired what potential avenues exist for the enhancement of preclinical training in calculus removal utilizing ultrasound devices. However, no mention was made regarding the utilization of the Simodont dental trainer. As anticipated, a plethora of recommendations were proffered; however, a subset of respondents interpreted the query as “What enhancements could be made to the Simodont dental trainer to optimize preclinical training?” Accordingly, the responses are categorized into two distinct classifications: “general improvements” and “Simodont improvements.” In the section entitled “General Improvements,” the faculty members recommended a combination of conventional simulation practices with Simodont dental trainers (4 mentions), an increase in the number of practical classes with Simodont dental trainers (2 mentions), and an increase in the number of practical classes with conventional ultrasound devices (1 mention). With respect to the concept of “Simodont improvements,” the responses exhibited a greater diversity of opinion. The recommendations to enhance the general realism of the exercise, to expand the array of dental models, and to incorporate subgingival calculus were each mentioned on three occasions. Two recommendations were proposed for the limitation of the model's mobility, the enhancement of indirect vision, and the incorporation of sensations, as well as the improvement of tactile calculus sensation. Finally, with one recommendation each, there were the introduction of behaviors/movements, the capacity to utilize the mirror and the probe concurrently, the enhancement of pressure indicators, the implementation of water, aspiration, and bleeding, the incorporation of various colors for calculus and dental stains, the introduction of anesthetic techniques, and the overall refinement of the device.

Finally, the fourth question posed to the participants sought their opinion on the optimal timing for the Simodont dental trainer exercise, namely whether it should precede or follow the conventional simulator exercise. The preponderance of respondents, amounting to 23, expressed a predilection for undertaking the Simodont exercise prior to the conventional simulator exercise. Conversely, four faculty members indicated a preference for the Simodont exercise following the conventional simulator exercise, while two stated no preference.

### 3.5 Calibration of examiners

Before data collection, two independent examiners (J.F. and Z.C.) were trained and calibrated using pilot cases on the Simodont® dental trainer, applying the study variables (time, sonic tip positioning, and amount of calculus removed). Inter-examiner reliability was assessed with Cohen's kappa coefficient, which yielded a value of 0.87, indicating excellent agreement. Intra-examiner reliability was verified by re-evaluating 20% of the cases after a two-week interval, confirming high consistency. All assessments were therefore considered reliable for subsequent analyses.

## 4 Discussion

Preclinical training in dentistry focuses on developing diagnostic and technical competencies through simulated practice (28). Traditionally, this has involved conventional simulators and direct faculty oversight. However, these methods often rely on subjective assessment and limited feedback (29). This study evaluated faculty performance and perceptions when using a virtual reality haptic simulator (VRHS) for ultrasonic scaling compared with conventional mannequin-based simulation, focusing on objective outcomes (treatment time, residual calculus, and tip angulation) and structured qualitative feedback, thereby addressing an underexplored domain in preclinical periodontics from the viewpoint of instructors who design and assess training.

### 4.1 Treatment time

Procedures with conventional simulation were completed faster, with most finished within 2 min, while longer times occurred only in the VRHS group. This cannot be attributed to lack of user familiarity, as all participants met competence thresholds beforehand. The longer duration probably results from the early developmental stage of the VR module used. Rodrigues et al., found similar patterns (initial tasks in VR-haptic simulators took longer but improved with practice), suggesting that efficiency increases over time as both users and technology advance (30). This highlights the need for ongoing collaboration to improve haptic software and feedback algorithms, balancing accuracy and efficiency. As periodontal modules advance with better calibration and realism, task duration should match or surpass conventional simulation It must be also taken into consideration that immediate feedback in VRHS environments enables operators to accurately assess task completion, which encourages them to thoroughly remove calculus before ending the procedure. This increased attention to detail may also result in longer overall treatment times.

There were no significant differences observed in treatment time between periodontists and general dentists. This convergence is unsurprising, as sonic scaling is routinely performed by both groups in daily practice. Ben-Gal et al. previously demonstrated significant differences in task duration among students, dentists, and non-dentists when assessing manual dexterity, suggesting that performance gaps are mainly associated with general clinical experience rather than specialization (31). In the present study, all participants were qualified dental professionals—either specialists or general practitioners—hence the absence of significant intergroup variation in timing outcomes.

### 4.2 Residual calculus

Residual calculus scores were higher in VRHS compared to conventional instrumentation, indicating more complete calculus removal. These results align with Fu et al., who reported lower residual calculus using VR-haptic systems than traditional methods, highlighting the precision afforded by haptic feedback and real-time monitoring (20). In VRHS, operators continuously visualize progress, adjust tip positioning and pressure, and verify thorough cleaning before ending the task, unlike conventional simulators, which depend on *post-hoc* examiner evaluation. The apparent superiority of VRHS reflects its feedback-rich learning loop and high detection sensitivity, which may also reveal subtle deposits overlooked by conventional assessments. Educationally, this environment encourages meticulousness, accurate tactile control, and greater learner autonomy. However, as commented before, longer procedure times suggest a trade-off between accuracy and efficiency, underscoring the need for software refinement to calibrate sensitivity, realism, and workflow speed, which is consistent with findings by Murbay et al. and Al-Saud et al. (32, 33). Ongoing technological optimization and alignment with standardized clinical benchmarks are crucial to improve ecological validity and clinical skill transfer (29). Overall, the present findings support the pedagogical value of VRHS as a feedback-intensive training tool that enhances accuracy and autonomy in psychomotor learning, while simultaneously emphasizing the need for iterative refinement of the periodontal module to achieve optimal realism and temporal efficiency.

When comparing performance between professional groups, no significant differences in residual calculus were observed between periodontists and general dentists, despite the overall superiority of VRHS compared with conventional simulation. This convergence between professional profiles suggests that the advantages conferred by the VR-haptic system—particularly its automated feedback, enhanced visualization, and objective scoring—act as a performance equalizer, minimizing the influence of prior specialization or clinical focus.Other studies such as that of Kropmans et al., which evaluated probing depth assessment in students and trainees, or Ben-Gal et al., who compared manual dexterity among students, dentists, and non-dentists, reported clear differences between groups (31, 34). Those studies examined cohorts at markedly different stages of clinical maturity, whilein the present work, all participants were experienced dental educators routinely performing periodontal instrumentation, which likely reduced intergroup variability. These findings are consistent with previous reports indicating that VRHS platforms tend to standardize psychomotor performance across users with different experience levels, owing to their objective metrics and automated feedback (17). Similar results have been reported in other studies, which found that periodontal instrumentation tasks performed by experienced clinicians show minimal intergroup variability regardless of specialization (31, 32, 35). Moreover, several authors have emphasized the potential of VR-haptic systems as calibration tools for faculty training and performance harmonization (36, 37).

### 4.3 Tip angulation

For tip angulation, VRHS was also associated with significantly higher scores compared with conventional training. This improvement can be attributed to the simulator's enhanced visualization field, prompting more deliberate control of stroke direction and instrument positioning. Such feedback mechanisms foster fine motor awareness and favour the acquisition of ergonomic precision—skills that are often difficult to assess objectively in conventional settings. Within the VRHS condition, periodontists achieved significantly higher angulation scores than general dentists, whereas no significant difference was detected in the conventional modality. This pattern was expected, as specialists possess refined spatial perception and greater familiarity with the delicate alignment required in periodontal instrumentation. However, the absence of group differences in the conventional simulator suggests that both profiles perform similarly under manual, experience-driven conditions where evaluation relies on examiner judgment. These findings again align with previous work, who reported improved precision and control of instrument angulation when using VR-haptic systems (20). Together with the results on residual calculus and treatment time, the current data indicate that VRHS encourages operators to prioritize accuracy and controlled movement over speed, reinforcing safe and consistent technique. From an educational perspective, this ability to quantify micro-movements and highlight differences in fine control demonstrates the pedagogical potential of VR-haptic platforms to both differentiate and standardize psychomotor training. Continued refinement of software fidelity—particularly regarding tactile calibration and real-time visualization—will further enhance their capacity to replicate the nuances of periodontal instrumentation and optimize learning efficiency.

### 4.4 Quantitative responses to the questionnaire

When analysing the quantitative responses, no statistically significant differences were observed between periodontists and general dentists across any of the thematic blocks. Both groups reported high satisfaction and confidence with VRHS, agreeing it supports motor-skill acquisition and learners self-assurance for sonic scaling. Faculty also valued its safe, repeatable environment with real-time performance feedback.

In contrast, perceptions of realism were more variable. Participants agreed that tooth morphology, calculus hardness, and tactile response were acceptably reproduced, but differed on overall similarity to conventional simulators. About half rated the sensation as “very similar,” while the remainder were neutral or disagreed, echoing ongoing debate about haptic realism. Prior studies have criticized limitations in motion, vibration, and sensory feedback; the more positive views here may relate to the newer periodontal module with improved algorithms (17, 19, 20).

Despite realism concerns, there was strong consensus that VRHS is a valuable adjunct to preclinical training, improving fine motor control, confidence, and independent learning. Many advocated integrating VRHS into basic periodontology curricula within hybrid models. These perceptions are consistent with prior studies demonstrating the pedagogical benefits of integrating VRHS into preclinical education to enhance psychomotor learning and motivation (32, 38).

Most participants advocated for the integration of VRHS exercises into the basic periodontology curriculum, This aligns with other studies, which supported hybrid learning models combining virtual haptic and conventional simulation to optimize skill acquisition and transfer to clinical practice (10, 19, 20, 30, 39).Opinions on fully replacing conventional simulation were divided, reflecting differing comfort levels with emerging technologies (10, 20).

Prior studies collecting student feedback, have shown greater acceptance of full substitution, largely due to the objectivity and consistency of VRHS-based assessment, which eliminates examiner bias and supports self-directed learning (20, 40). In contrast, experienced faculty—accustomed to manual assessment protocols—may retain a more cautious attitude until future iterations of haptic modules incorporate more realistic multimodal feedback (e.g., water spray, tissue compliance, or indirect vision).

Overall, faculty perceived VRHSas effective and promising, while emphasizing the need for continued refinement of software fidelity and tactile realism to better align virtual training with clinical practice.

### 4.5 Qualitative responses to the questionnaire

The qualitative responses to the open-ended questions contextualize the faculty's quantitative feedback. Educators widely acknowledged the pedagogical value of the Simodont dental trainer, especially its capacity for repetition and its contribution to confidence and perceived safety yet consistently highlighted important technical and sensory limitations. This dual perception reflects findings by Bakr et al. indicating that, indespite technical progress, concerns about realism and tactile authenticity remain influential in decisions about curricular integration (24).

The main limitation reported in our study was insufficient, frequently linked to missing features such as water, soft tissues, subgingival calculus, blood, saliva, texture, aspiration, or patient interaction. These concerns parallel previous work showing inadequate tactile discrimination and hardness differentiation in VR-haptic systems (19). The convergence of faculty and student opinions across contexts suggests that these issues stem from intrinsic challenges in reproducing authentic haptics rather than isolated device shortcomings.

Several respondents recommended combining conventional simulation with VR-haptic training, viewing VRHS as a complement rather than a replacement. This aligns with evidence supporting hybrid models that unite tactile realism, objective feedback metrics, and feedback-driven learning (19). Participants also proposed practical enhancements—improved tactile fidelity, expanded periodontal and restorative scenarios, realistic indirect vision, and integration of water and aspiration systems—offering a clear roadmap for developers.Notably, many identified limitations mirror those reported over a decade ago (18), despite advances in interface design and visualization (17, 19). This persistence underscores the need for targeted innovation in multisensory and force-feedback technologies to achieve immersive, clinically transferable experiences.

Finally, participants emphasized expanding access and practice time with both conventional and VR-haptic platforms. They envisaged these tools as integrated components of a broader educational ecosystem, supporting progressive skill acquisition from preclinical to clinical stages. Overall, the qualitative data reinforce that the optimal role of VRHS in dental education lies in thoughtful integration within blended curricula, where it strengthens, rather than substitutes, the experiential richness and tactile reliability of traditional training (32).

### 4.6 Limitations and future directions

This study has certain limitations that should be considered. Perception-related outcomes were self-reported, which may introduce response bias, and the single-institution design limits generalizability to other academic or cultural settings. Although participants were homogeneous in background and all achieved ≥75% VRHScalibration competence, this methodological strength for internal consistency also reduces external validity. The sample size (*n* = 30) was modest, constraining statistical power for subgroup comparisons. Moreover, only short-term performance and perceptions were evaluated; long-term skill retention, transfer to clinical contexts, and learning outcomes were not assessed. The results should therefore be interpreted within the scope of immediate pedagogical impact. Additionally, the periodontal module employed remains under technological refinement, particularly regarding force calibration, tactile realism, and integration of soft-tissue dynamics, which may have influenced performance times and realism ratings. The study was further constrained by the Simodont® Periodontology module, which currently allows instrumentation only on mandibular incisors (32–31–41–42) and by the exclusive use of a single VR-haptic platform available at the institution. This focus on a single simulator was intentional, as the study coincided with the release of the updated Simodont® Periodontology module, which required expert faculty evaluation prior to potential curricular integration. Nonetheless, the use of only one VR-Haptic system (Simodont®) represents a limitation. Future investigations comparing multiple simulators, such as SimToCare® or Unidraw®, would provide broader insight into potential differences in haptic feedback, educational design, and competency development. This limited anatomical range restricts the ecological validity of the task and precludes broader assessment across posterior regions. The results should therefore be interpreted within this specific context. Future VR-haptic systems, such as SimToCare® and UniDraw®, which incorporate full-arch periodontal modules, may offer enhanced training potential but should be carefully tested and validated by experienced dental educators prior to curricular integration.

Moreover, manual examiner-based scoring used in the conventional simulation group may introduce a degree of subjectivity and inter-rater variability. Future research should therefore consider implementing machine-based, hybrid human–machine, or AI-assisted assessment systems to enhance objectivity, reproducibility, and scalability in evaluating psychomotor performance.

Future research should include multicenter and longitudinal designs, objective performance analytics, and further evaluation of technological and pedagogical enhancements to optimize realism, feedback precision, and clinical transferability. Continued collaboration between academia and industry will be essential to strengthen the educational value and scalability of VRHSin preclinical dental training.

## 5 Conclusion

Within the limitations of this preliminary study, the integration of VRHSproved to be an effective adjunct to conventional preclinical training in periodontics. Faculty participants perceived it as a valuable tool for enhancing precision, control, and confidence in sonic scaling, with no major differences between specialists and general dentists. Although procedures required longer times and some limitations in tactile realism were noted, the simulator's educational benefits—repetition, feedback, and objectivity—were widely recognized. Rather than replacing conventional simulation, VR-Haptic training should be regarded as a preparatory and complementary stage that facilitates the correction of angulation, handpiece position, and movement control prior to clinical or mannequin-based practice. Continued technological refinement and broader validation are needed to consolidate VRHSas a standardized component of periodontal education.

## Data Availability

The original contributions presented in the study are included in the article/Supplementary Material, further inquiries can be directed to the corresponding author.
